# The epidemiology of snakebites, treatment-seeking behaviour, and snakebite management in the department of Ogooué et des Lacs, Gabon, Central Africa: a cross-sectional community and health facility-based survey

**DOI:** 10.7189/jogh.15.04062

**Published:** 2025-04-25

**Authors:** Rica Artus, Jade Rae, Friederike Hunstig, Ghyslain Mombo-Ngoma, Alex Hounmenou Zinsou, Dearie Glory Okwu, Wilfrid Ndzebe Ndoumba, Rella Zoleko Manego, Michael Ramharter, Bertrand Lell, Peter Gottfried Kremsner, Moses Banda Aron, Jörg Blessmann, Benno Kreuels

**Affiliations:** 1 Research Group Neglected Diseases and Envenoming, Bernhard Nocht Center of Tropical Medicine, Hamburg, Germany; 2 Centre de Recherches Médicales de Lambaréné, Lambaréné, Gabon; 3 Division of Infectiology, Outpatient Center, University Medical Center Hamburg-Eppendorf, Hamburg, Germany; 4 Research Group Drug Implementation, Department of Implementation Research, Bernhard Nocht Institute for Tropical Medicine Hamburg, Germany and I. Department of Medicine, University Medical Center Hamburg-Eppendorf, Hamburg, Germany; 5 Center for Tropical Medicine, Bernhard-Nocht Institute for Tropical Medicine and I. Department of Medicine, University Medical Center Hamburg-Eppendorf, Hamburg, Germany; 6 German Center for Infection Research (DZIF), Partner Site Hamburg-Luebeck-Borstel-Riems, Hamburg, Germany; 7 Institute of Tropical Medicine, Eberhard Karls University, Tübingen, Germany; 8 Division of Infectious Diseases and Tropical Medicine, I. Department of Medicine, Medical University of Vienna, Vienna, Austria; 9 German Center for Infection Research (DZIF), Partner Site Tübingen, Tübingen, Baden-Württemberg, Germany; 10 Partners In Health/Abwenzi Pa Za Umoyo, Neno, Malawi; 11 Division of Tropical Medicine, Department of Medicine I, University Medical Center Hamburg-Eppendorf, Hamburg, Germany

## Abstract

**Background:**

Snakebite envenoming is a neglected public health problem in many tropical countries, resulting in over 100 000 deaths and 400 000 disabilities worldwide each year. In Gabon, where venomous snakes are abundant, studies on the epidemiology and treatment of snakebites are lacking.

**Methods:**

Between October 2022 and June 2023, we conducted a cross-sectional community survey in the department of Ogooué et des Lacs in central Gabon to estimate the snakebite incidence, describe clinical presentations and treatment-seeking behaviours, and describe the burden of snakebites to animal populations in rural and urban communities. We also surveyed health facilities in the department to describe treatment practices and the availability of antivenom.

**Results:**

The standardised annual incidence rate was 246 snakebite cases per 100 000 person-years (95% confidence interval (CI) = 138–438). Of the 175 snakebite cases reported in the five years prior to the survey, 18% showed signs of envenomation, predominantly with cytotoxic signs. The mortality among the bitten population was 3%. Snakebite treatment was first sought at a formal health facility in 55% of cases, from traditional healers in 22%, and with self-treatment or no treatment in the remaining 23%. Of snakebite patients treated at a formal health facility in the five years prior to the survey, 81% received antivenom, 41% received antibiotics, and 51% received corticosteroids. Almost one in six households reported animal deaths due to snakebites in the previous 12 months.

**Conclusions:**

This study provides the first robust epidemiological estimates of the burden of snakebites in Gabon and highlights the importance of community-based surveys in accurately assessing this high burden. Training health care workers, developing treatment guidelines, and ensuring the availability of effective and affordable antivenom are important steps to improving the outcome for snakebite victims.

In 2017, the World Health Organization (WHO) added snakebite envenoming to the list of neglected tropical diseases and proposed a strategy to reduce the mortality and morbidity of snakebite envenoming by 50% by 2030. This strategy focusses on four main areas: safe and effective treatment, empowering and engaging with communities, strengthening health systems, increasing partnerships, and improving coordination and resources [1].

For this strategy to be effective, resources should be directed to areas where the burden of snakebite is highest. This tends to be in rural areas of low-income countries in Asia, Africa, and Latin America due to a greater risk of snake-human interactions and limited access to effective treatment [2–4]. However, estimates of snakebite burden at a fine geographic scale are often lacking, and incidence estimates that have relied on health facility data alone are thought to underestimate the true burden, as only a small proportion of snakebite victims seek treatment within the formal health care system [2,5,6]. This is due to the ongoing preference for traditional medicine, the high out-of-pocket cost of treatment at health facilities, poor supplies and knowledge of health care workers [7-9], and the long distances required to travel to health facilities from rural areas [7,10–12].

A significant proportion of snakebites result in mild or no clinical signs, either because the snake is non-venomous or because little or no venom is injected. These cases do not require clinical intervention. However, the injected venom of some snake species can cause local tissue damage (cytotoxic effects), life-threatening effects such as paralysis of the respiratory muscles (neurotoxic effects), or severe bleeding (haematotoxic effects). Patients suffering from severe envenoming urgently require antivenom and supportive care [13,14]. To improve the clinical outcomes following envenomation, it is essential to educate health care workers and expand the availability of essential resources [7–9].

Snakebite envenoming is also associated with a significant financial burden due to the high out-of-pocket cost of treatment at health facilities and the inability of snakebite victims or caregivers to work during recovery. Additionally, high mortality rates among bitten domestic animals [15,16] further exacerbate the financial burden [17], particularly among populations that rely on domestic animals for their income.

Gabon, a country in Central Africa, is home to at least 30 species of venomous snakes [18], five of which are classified by the WHO as being of highest medical importance for which antivenom should be locally available, due their frequent presence in inhabited areas and their potential for life-threatening envenomation. These include *Bitis gabonica, Bitis nasicornis, Naja nicgricollis, Dendroaspis jamesoni*, and *Naja melanoleuca* [19]. Studies in Gabon to date have been limited to a 2002 retrospective analysis of snakebite cases admitted to a hospital in the capital, Libreville [20], and a small pilot study in one rural village [21]. To better understand the snakebite burden and identify gaps in the management and treatment of snakebite victims in Gabon, we conducted a community-based survey and a retrospective analysis of cases admitted to health facilities in the department of Ogooué et des Lacs in the province of Moyen-Ogooué, in central Gabon.

## METHODS

### Study design

We conducted a household-based cross-sectional survey in the department of Ogooué et des Lacs, Gabon, between October 2022 and June 2023 to determine the incidence of snakebites and describe the treatment-seeking behaviours, symptoms, and clinical outcomes for those bitten. Additionally, we retrospectively extracted data on snakebite patients treated at health facilities in the department between October 2017 and June 2023 to determine treatment practices and assess the availability and type of antivenom in these facilities. We reported our findings according to STROBE guidelines (**Online Supplementary Document**).

### Study area

Gabon has nine provinces subdivided into 49 departments. Ogooué et des Lacs is one of the two departments of the Moyen-Ogooué province in central Gabon. It is a lowland area, with dense rainforests covering approximately 80% of the department, and there are typically two annual rainy seasons [22,23]. The estimated population from the last census in 2013 was 54 346, with the majority (38 755) living in Lambaréné, the capital of the province [24]. The main economic activities include subsistence farming, fishing, hunting, and work on palm oil plantations.

### Community survey

#### Sample size calculation

Based on a pilot study conducted in 2021, we assumed an annual incidence of at least 100 snakebites per 100 000 inhabitants [21]. To estimate this incidence with 95% confidence interval (CI), a precision of ±75 cases per 100 000 person-years, and a design effect of 1.5 to adjust for clustered sampling of individuals within households, the estimated required sample was 10 165 respondents. We calculated this sample size using OpenEpi, version 3.01 [25]. Based on an average household size of 3.5 people (estimated from the Health and Demographic Surveillance System) implemented in 2016 by the *Centre de Recherches Médicales de Lambaréné* (CERMEL)) [26], and a potential non-response rate of 10% (approx. 300 households), we aimed to survey 3200 households.

#### Household and participant selection

**Figure 1:**
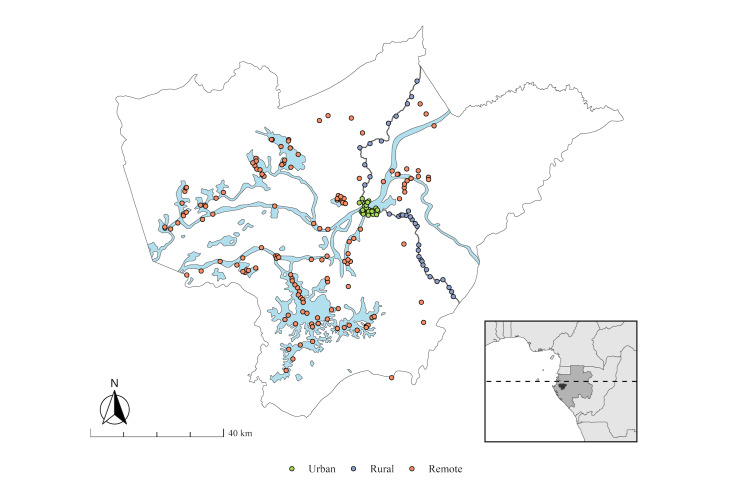
Communities included in the cross-sectional survey in the department of Ogooué et des Lacs, Gabon. Each point represents a surveyed community in the urban (green), rural (dark blue), and remote (red) sectors. The blue areas represent the lakes and the Ogooué River, and the grey line represents the main road. The inset map shows the locations of Ogooué et des Lacs (black), Gabon (dark grey), and other countries in the region, with the Equator shown as a dashed line.

We divided the department into three sectors based on accessibility, geography, and administrative structures. The urban sector encompassed the town of Lambaréné; the rural sector consisted of villages along the main road that runs through the department from north to south; and the remote sector consisted of camps and villages on unpaved roads in the forest and around the lakes and riversides which are only accessible by four-wheel-drive vehicles or boat (**Figure 1**).

Due to the lack of a sampling frame in the rural and remote sectors, we decided to conduct a complete surveying of the rural and remote population by interviewing all households within each community. The remaining households required to reach the target sample size were randomly selected from the urban sector in Research Randomizer, version 4 [27] using the list of households within the Health and Demographic Surveillance System as the sampling frame. We scheduled surveys in the early morning or late afternoon to increase survey participation. We excluded households if they were uninhabited, inhabited by people who do not permanently reside in the household (*e.g.* vacation houses), or if residents were not encountered after two visits by the study team.

#### Patient and public involvement

We considered patient and public involvement from the cross-sectional survey, whereby we adapted the questions within the survey after it was piloted in the communities of Gabon to ensure that questions were relevant, acceptable, and easy to understand.

#### Data collection

We used a structured questionnaire for the conduction of the survey (**Online Supplementary Document**). We collected the age and sex of all household members and information on domestic animals owned by the household. If a snakebite was reported, we collected additional information. If an individual reported multiple snakebites, we collected information for each incident separately. Information on snakebites that resulted in death was collected from someone who was with the bitten individual during or after the incident. In several instances, individuals who did not permanently reside in one of the surveyed households approached the study team to report snakebites. We included them as supplementary respondents and collected data on their age, sex, and the reported bite. If a household owned animals in the previous 12 months, we recorded any information on snakebites to the animals. Data were collected and managed Research Electronic Data Capture (REDCap) tools, including the mobile application and the web platform [28,29].

### Health facility survey

#### Health facility selection

We identified and surveyed 28 formal health facilities, including two regional hospitals and 26 primary health facilities (21 dispensaries, four medical centres, and one outpatient clinic). We also included one pharmacy to assess antivenom supplies.

#### Data collection

We retrieved data on snakebite patients treated between October 2017 and June 2023 from patient records at the health facilities. These included the patient’s age and sex, details on when and where the bite occurred, symptoms following the snakebite, treatment received, diagnostic tests, health facility admission, and clinical outcome. We additionally recorded the number and type of antivenom vials available at these facilities. These data were collected and managed using REDCap electronic data capture tools [28,29].

### Data management

We excluded snakebites where the bite was deemed implausible or if a sufficient description could not be provided to assess the plausibility using clarifying questions in the community survey. We retrospectively classified the severity and syndromes of envenoming using criteria consistent with the WHO guidelines and current literature (Table S1 in the **Online Supplementary Document**) [14,30]. If the snake was seen after biting a human or domestic animal, we retrospesctively assigned a possible species using a combination of the reported colour and name in line with the species common in the study area [18,31,32]. Snakebites to domestic animals were excluded if the snake was not seen. We cleaned and analysed the data in *R*, version 4.3.1 (R Core Team, Vienna, Austria) [33].

### Statistical analysis

#### Incidence calculations

We included only cases reported by the surveyed household residents in the previous 12 months in the incidence calculation. We then calculated the incidence of snakebites for the three sectors separately as the number of snakebites over person-time exposed, and person-time exposed as the total number of household members surveyed in each sector who provided information on whether they were bitten by a snake in the previous 12 months. We calculated the standardised incidence rate to provide a single incidence estimate for the department, while considering the different sampling procedures between the sectors. To do this, we determined a weighting factor for each sector by dividing their total estimated population by their sampled population. Then, we multiplied each snakebite case by the weighting factor of their corresponding sector, and obtained the standardised incidence per 100 000 persons per year as the sum of these weighted case numbers over the total estimated population in the department.

We calculated the incidence of snakebites from health facility data as the total number of department residents presenting to a health facility following a snakebite in the previous 12 months over person-time exposed, where person-time exposed was the estimated population in the department.

To calculate the weighted incidence and health facility incidence, the estimated population was taken from the last census for the urban sector (n = 38 755) and was collected during the survey for the rural (n = 5672) and remote (n = 4749) sectors. The 95% CIs for all incidence calculations were computed as Wald-type intervals using the ‘srvyr::survey_mean’ package in *R* [33,34].

#### Description of snakebites to humans

We used descriptive statistics (means and ranges, medians (MDs) and interquartile ranges (IQR), and frequencies and percentages) to describe snakebites reported in the community survey. To reduce the effect of recall bias on the summary statistics, we described only cases in the previous five years. We recorded the reported cost of treatment in the local currency (XAF) and converted it to USD using the average exchange rate in the year of the bite [35].

We described snakebite cases extracted from health facility records using MDs (IQRs) or frequencies and percentages. Snakebites reported in the community survey and patient records with missing data for certain variables were included in the analysis, but excluded from calculations for those variables, resulting in different denominators.

#### Snakebites to domestic animals

We summarised the number of herds owned by the surveyed households and the frequencies and percentages of herds bitten by a snake in the previous year using descriptive statistics. We defined a herd as a group of animals of defined species owned by a household (*e.g.* two chicken herd bites = at least one chicken bitten among the herds of chickens owned in two households) [30].

We calculated the incidence of animal bites for each species separately as the number of herd bites over the number of households owning a herd of that animal, and the mortality rate as the number of herd bites resulting in at least one death over the number of herds bitten of that animal.

## RESULTS

### Community survey study population

We approached 3215 households during the cross-sectional survey. Of these, 133 (4.1%) were uninhabited and 87 (2.7%) were vacant at the time of visit. Of the remaining 2995 households, 75 (2.5%) refused to participate, and 11 (0.4%) were excluded because no information on the household or household members was collected. Therefore, we included 2909 households and 12 480 household members in the survey (Figure S1 in the **Online Supplementary Document**), of which 1281 were in the rural sector (5477 individuals), 1196 were in the remote sector (4530 individuals), and 432 were in the urban sector (2473 individuals).

### Incidence of snakebites in the last 12 months

Forty-six snakebites were reported by the surveyed household members in the 12 months prior to the survey, corresponding to a standardised incidence rate of 246 snakebites per 100 000 person-years (95% CI = 138–438). By sector, the incidence rate was highest among the rural population, but this did not differ significantly from the incidence in the remote or urban populations, as shown by the overlapping 95% CIs (**Table 1**).

**Table 1:** Community-based snakebite incidence in the previous 12 mo by sector

	Not bitten, n (%)	Bitten, n (%)	Incidence per 100 000 person-years (95% CI)
Urban	2468 (99.8)	5 (0.2)	202.2 (84.1–485.1)
Rural	5453 (99.6)	24 (0.4)	438.2 (293.8–653.0)
Remote	4513 (99.6)	17 (0.4)	375.3 (233.4–602.9)
Overall*	12 434 (99.6)	46 (0.4)	246.1 (138.1–438.1)

CI – confidence interval

*Overall incidence is calculated as the standardised incidence rate across sectors.

### Circumstances of snakebites

In the five years prior to the survey (**Table 2**), 176 snakebite incidents were reported, 150 by household members and 22 by supplementary respondents. These bites occurred in 172 individuals, with two being bitten twice and one three times. Males accounted for 59.7% (n/N = 105/176) of snakebite cases, and the median age at the time of the bite was 42.5 years (IQR = 26–54). Exactly half (n/N = 88/176) of all reported snakebites occurred during daytime, 15.9% (n/N = 28/176) at dusk and 34.1% (n/N = 60/176) at night. The majority of snakebites occurred in a forest or plantation (n/N = 79/176, 44.9%), inside (n/N = 16/176, 9.1%) or around (n/N = 34/176, 19.3%), the house, or at the riverside (n/N = 26/176, 14.8%). Bites mainly occurred while people were walking (n/N = 75/175, 42.9%), doing manual agricultural work (n/N = 36/175, 20.6%), or hunting (n/N = 28/175, 16.0%), and were mostly to the lower limbs (n/N = 132/175, 75.4%).

**Table 2:** Characteristics of snakebites and clinical consequences

	n (%)
**Total snakebites**	176*
**Location**	
Forest/plantation	79 (44.9)
Yard/garden	34 (19.3)
Riverside	26 (14.8)
Footpath or road	18 (10.2)
House (inside)	16 (9.1)
Village (public place)	3 (1.7)
**Activity**†	
Walking	75 (42.9)
Manual agricultural work	36 (20.6)
Hunting	28 (16.0)
Fishing	10 (5.7)
Bathing	8 (4.6)
Other	18 (10.3)
**Body part bitten**†	
Lower limb	132 (75.4)
Upper limb	40 (22.9)
Trunk/head/neck	3 (1.7)
**Classification of snakebite severity**†‡	
Dry/mild	143 (81.7)
Moderate	18 (10.3)
Severe	14 (8.0)
**Syndromes of severe envenomation**	
Cytotoxic	7 (4.0)
Neurotoxic	5 (2.9)
Neurotoxic and cytotoxic	1 (0.6)
Unclear	1 (0.6)
**Clinical outcome**	
Physical recovery	165 (93.8)
Physical impairment	6 (3.4)
Death	5 (2.8)

*In the previous five years, three individuals were bitten multiple times (two individuals twice, one individual three times), resulting in 176 snakebite cases in 172 individuals.

†Information missing for one case, resulting in a denominator of 175.

‡Classification was determined retrospectively based on the criteria detailed in Table S1 in the **Online Supplementary Document**.

The snake responsible for the bite was seen in 84.1% (n/N = 148/176) of cases, and a possible snake species could be assigned in 13.1% (n/N = 23/176) of all cases. Of these, *N*. *melanoleuca* was assigned in nine cases, *P. sebae* in eight cases, and *B. gabonica* in six cases (Table S2 in the **Online Supplementary Document**).

### Symptoms and classification of snakebite severity

According to our classification criteria, dry bites and mild envenomations were the most common (n/N = 143/175, 81.7%), followed by moderate (n/N = 18/175, 10.3%) and severe envenomations (n/N = 14/175, 8.0%). Snakebites classified as severe exhibited signs of cytotoxicity (n/N = 7/175, 4.0%), neurotoxicity (n/N = 5/175, 2.9%), or cytotoxicity and neurotoxicity (n/N = 1/175, 0.6%). One individual (n/N = 1/175, 0.6%) with an unclassified syndrome died following the snakebite (**Table 2**; Table S3 in the **Online Supplementary Document**).

### Treatment-seeking behaviour and treatment received

After a snakebite, most surveyed individuals first visited a health facility (hospital: n/N = 63/175, 36.0%; primary health facility: n/N = 33/175, 18.9%), and 22.3% (n/N = 39/175) first visited a traditional healer. Overall, 22.9% (n/N = 40/175) did not seek treatment from a health care provider, primarily those living in the rural (n/N = 19/89, 21.3%) and remote (n/N = 18/60, 30.0%) sector. Of those who did not seek treatment from a health care provider, 65% (n/N = 26/40) self-administered traditional remedies (Table S4 in the **Online Supplementary Document**).

**Figure 2:**
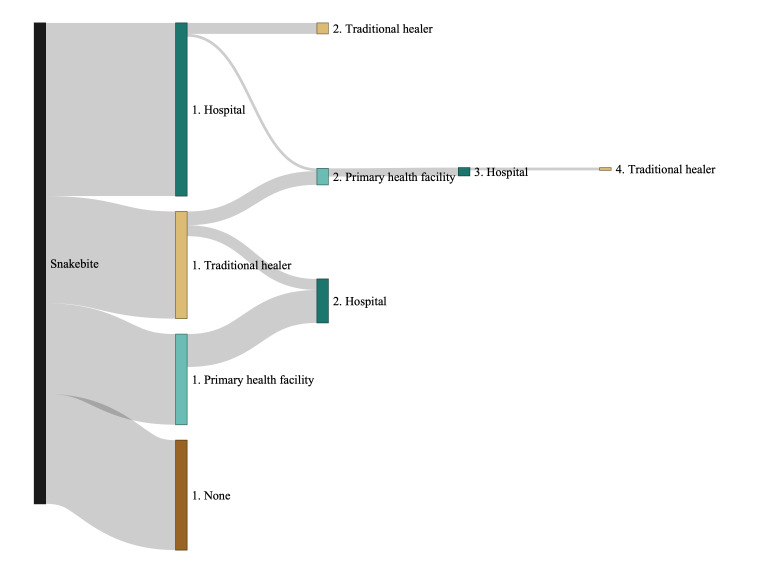
Treatment-seeking behaviour following a snakebite. Details on snakebite treatment were collected retrospectively for cases between October 2017 and June 2023 in Ogooué et des Lacs. This figure shows data collected from 175 snakebite cases (information was missing for one case).

Of the 33 individuals who first visited a primary health facility, 12 (36.4%) were subsequently transferred to a hospital. In comparison, only five of the 39 (12.8%) patients who first visited a traditional healer were subsequently seen in a primary health facility, and only four (10.3%) were subsequently transferred to a hospital (**Figure 2**). Out of 63 patients that first presented to a hospital, four (6.3%) subsequently went to a traditional healer and one out of 63 (1.6%) to a primary health facility. One patient visited four different health care providers, first a hospital, then a primary health facility, followed by a hospital, and finally a traditional healer.

Of the 105 individuals who visited a health facility after being bitten, 84 (80.0%) reported receiving antivenom. By severity, antivenom was administered to 80.3% (n/N = 61/76) of cases classified as dry/mild, 87.5% (n/N = 14/16) classified as moderate, and 69.2% (n/N = 9/13) classified as severe. Six of the 105 individuals (5.7%) reported that a device called ‘Aspivenin’ [36] or ‘Venimex’ [37] was used in an attempt to extract snake venom from the wound. Traditional treatment (including bark, leaves, or herbs) was administered in 62.3% (n/N = 109/175) of cases and was applied topically (n/N = 97/109, 89.0%), and/or taken orally (n/N = 53/109, 48.6%). The application of a tourniquet was reported by 50.9% (n/N = 89/175) of bitten individuals.

### Clinical outcomes following snakebites

Five (2.8%) out of 176 individuals died following a snakebite. All were female and over the age of 50. Three exhibited signs of neurotoxic envenomation, one exhibited signs of cytotoxic envenomation, and one could not be classified. One self-administered traditional medicine and died at home; one visited a traditional healer, followed by a primary health facility, before arriving at a hospital and receiving antivenom; one visited a primary health facility before arriving at a hospital and receiving antivenom; and two went directly to a hospital, where one died on arrival, and the other reportedly received antivenom before dying.

A total of 165 patients (93.8%) fully recovered, while six (3.4%) reported ongoing physical symptoms. These included fatigue (n/N = 1/6, 16.7%), cramps (n/N = 2/6, 33.4%), pain (n/N = 2/6, 33.4%), swelling (n/N = 1/6, 16.7.%), and difficulty walking (n/N = 2/6, 33.3%).

### The economic impact of snakebites

The cost of treatment was provided in 51.1% (n/N = 69/135) of snakebite cases where treatment was sought. From these estimates, the median out-of-pocket cost of snakebite treatment was USD 132 (IQR = 32.1–165.0) or XAF 80 000 (IQR = 20 000–100 000). By treatment provider, the reported median out-of-pocket cost was higher for individuals who exclusively sought treatment at a health facility, at USD 139.1 (IQR = 64.1–165.0) or XAF 80 000 (IQR = 40 000–100 000), when compared with those who exclusively sought treatment at a traditional healer, where the median cost was USD 8.7 (IQR = 2.0–8.7) or XAF 5000 (IQR = 1250–5000).

### Snakebites to domestic animals

Of the 2899 households that provided information on animal ownership, 1403 (48.4%) owned at least one herd in the 12 months prior to the survey, the majority of which owned chickens (n/N = 1174/1403, 83.7%). Approximately one-third of households with animals (n/N = 450/1392, 32.3%) reported a snakebite to at least one herd in the previous 12 months, with 96.2% (n/N = 431/448) of the herd bites resulting in at least one death. While bites were less common among goats/sheep and dogs/cats, almost all bites to these herds also resulted in at least one death (**Table 3**).

**Table 3:** Herd ownership and snakebite cases among domestic animals owned in the previous 12 mo by the surveyed households

Herd	Number of households with a herd*	Number of herd bites†	Herd bite incidence per 100 herds (95% CI)	Number of herd bites resulting in at least one death	Mortality rate per 100 herds (95% CI)
Chicken	1174	418	35.6 (32.9–38.4)	403‡	96.9 (94.7–98.2)
Goat/sheep	112	15	13.4 (8.3–20.9)	15	100.0 (79.6–100.0)
Dog/cat	584	43	7.4 (5.5–9.8)	37	86.0 (72.7–93.4)
Other§	58	2	3.4 (1.0–11.7)	2	100.0 (34.2–100.0)

*A herd represents a group of animals of the same species owned by a household.

†Multiple herd bites were reported by 28 households. The number of snakebites to animals was missing for 11 households which do not contribute to the incidence or mortality calculations.

‡Information on death following the snakebite was missing for two households which do not contribute to the mortality calculations.

§Other animals included ducks, monkeys, cows, and pigs.

A possible snake species could be assigned in 30.0% (n/N = 135/450) of bites to animals, of which 69.6% (n/N = 94/135) were *N. melanoleuca*, 29.6% (n/N = 40/135) were *P. sebae* and 0.7% (n/N = 1/135) were *B. gabonica* (Table S2 in the **Online Supplementary Document**).

### Evaluation of health facility data from snakebite patients and antivenom availability

In the 12 months prior to the survey, 29 snakebite patients were registered in health facility data, 23 of whom were Ogooué et des Lacs residents. Based on an assumed total population of 49 176 in the department, this corresponds to an incidence of 46.8 snakebites per 100 000 person-years (95% CI = 31.1–70.4) based on health facility data alone.

In the five years prior to the survey, 130 snakebite cases were recorded in the patient records at the visited hospitals (n/N = 119/130, 91.5%) and health facilities (n/N = 11/130, 8.5%). Of the 128 snakebite patients with available information on their place of residence, 79.7% (n/N = 102/128) were residents of the department.

Symptoms were recorded for 61.5% (n/N = 80/130) of snakebite patients, with the most common being pain (n/N = 32/80, 40.0%), swelling (n/N = 26/80, 32.5%), and vomiting (n/N = 11/80, 13.8%). Antivenom was administered to 80.7% (n/N = 96/119) of patients with data on treatment. In 1.6% (n/N = 1/64) of cases with available documentation, an allergic/toxic shock was reported. For the patients where the antivenom dose was recorded, 90.4% (n/N = 47/52) received one vial, and 9.6% (n/N = 5/52) received two vials. Other treatments included tetanus vaccination (n/N = 73/119, 61.3%), analgesics (n/N = 68/119, 57.1%; mainly paracetamol (n/N = 52/68, 76.5%)), corticosteroids (n/N = 61/119, 51.3%), antibiotics (n/N = 49/119, 41.2%) and local antiseptic wound treatment (n/N = 32/119, 26.9%). In addition, two patients developed a severe wound infection and necrosis requiring surgical debridement.

Of the 114 cases with admission details recorded, 72.3% (n = 83) were treated as inpatients. Length of stay was available for 66 inpatients, with a median length of one day (IQR = 1–2 days) and a maximum stay of 11 days for one patient. Details on clinical outcome was recorded for 66.3% (n/N = 55/83) of inpatients. Two inpatients were referred to hospitals in the capital of Gabon, Libreville. Three deaths were recorded among the inpatients in health facility data. All cases that resulted in death were also captured in the community survey.

Five of the 29 health facilities visited to assess antivenom availability were supplied with the polyvalent antivenom Inoserp PAN-AFRICA (Inosan Biopharma, Mexico/Spain), composed of equine-derived immunoglobulin fragments F(ab’)2 [38]. These health facilities included the two hospitals, the pharmacy in Lambaréné, and two remote sector medical centres, but no dispensaries. At the time of the visit, one medical centre had five vials, while the other health facilities stocked a maximum of three.

## DISCUSSION

This study provides the first robust estimate of the snakebite burden for Gabon with an estimated annual standardised incidence rate of 246 snakebites per 100 000 person-years. This estimate exceeds previous estimates for sub-Saharan Africa from a meta-analysis (204.6 cases per 100 000 people [6]), but is lower than those reported in rural populations for Cameroon (up to 665 per 100 000 [8]) and Kenya (up to 440 per 100 000 [39]). However, our estimates for the rural (438 cases per 100 000 person-years) and remote (375 cases per 100 000 person-years) sectors align with these estimates.

With an estimated mortality of 3% and assuming a similar distribution of snakebites around the country, this would extrapolate to 5994 cases and 180 deaths due to snakebite annually among Gabon’s 2.44 million inhabitants [40]. Assuming the population of Libreville, the capital [41], is not at risk for snakebites, this still results in 3820 cases and 115 deaths yearly. By comparison, rabies accounts for only 1–2 deaths annually [42]. These findings emphasise the significance of snakebite envenoming in Gabon, prompting its inclusion in the 2024–28 National Health Development Plan [43].

The higher-than-expected incidence in the urban sector reflects the environment of the town of Lambaréné, which has densely vegetated areas and water bodies throughout the city, providing natural habitats for snakes. Human activities may even increase the attractiveness of habitats for some snake species by attracting rodents and other prey and by facilitating the availability of food in and around houses (*e.g.* chicken) [31,32]. Further, the urban population is often engaged in agricultural activities and fishing, which increases the exposure to snakes and snakebites. Similar circumstances are common in many African urban areas, highlighting the need to include urban populations in snakebite surveys for accurate burden assessments.

The community survey revealed an incidence almost five-fold higher than the health facility data (47 cases per 100 000 person-years). This aligns with previous studies showing an underestimation of snakebite incidence in health facility data [7,8,12,39,44], as many victims do not seek treatment at formal health facilities. Further, incomplete records in the health facilities are common and case registers were available for the complete five-year observation period in both hospitals, but only in 5 out of 26 primary health facilities. The imminent implementation of the District Health Information System – 2 will include snakebite as a notifiable neglected tropical disease, potentially improving data completeness in the health facilities.

Another contributing factor could be an over-reporting of health facility attendance in the community survey due to a social desirability bias [45] because of anticipated negative attitudes toward traditional medicine among the study team. A meta-analysis on the use of traditional medicine in sub-Saharan Africa demonstrated that more than 50% of users do not disclose this to health care providers [46].

Over the past five years, 45% of reported snakebites in Ogooué et des Lacs occurred in a forest or plantation. This highlights the occupational risks of working in these environments, as observed in many other settings [3,7,8,13,47]. In addition, three-quarters of snakebites were to a lower limb, and approximately 20% of bites occurred in or around the home, predominantly during dusk or at night. These findings highlight the importance of prevention measures, such as clearing the area around houses, carrying a light when walking at night, and wearing protective footwear. Subsidising protective gear, particularly lamps, and establishing strict occupational health and safety standards for industrial plantations [48] could mitigate these risks. Community engagement campaigns to improve local knowledge of snakebite prevention have also been highly effective in several settings [49,50] and should be adapted and implemented in Gabon. Such initiatives must also address effective *vs.* harmful first-aid practices, such as the use of tourniquets [13,14,51,52], and appropriate treatment-seeking behaviours.

While the majority of individuals reportedly attended a health facility, similar to findings from Ghana and Kenya [39,53], approximately one-third of cases, primarily those living in the rural and remote sectors, sought traditional treatment only. It was uncommon for snakebite victims to seek treatment from both a traditional healer and a health facility. While there is no evidence to support the use of traditional treatment, as it may result in delayed antivenom administration and an increased risk of wound infection following the application of unsterile remedies to the bite site [54,55], sociocultural factors, accessibility, and lower costs, contribute to the continued reliance on traditional healers for the management of snakebite, especially in rural communities [11]. Therefore, training on effective first-aid and identification of moderate and severe envenomation that requires urgent referral to a health facility should be offered to traditional healers. Establishing collaborations between traditional healers and formal health facilities could ensure timely and effective treatment, improve outcomes and build trust between rural communities and the formal health system. Such a collaboration for Buruli Ulcer in Cameroon improved case detection and referrals through trust and collaboration between traditional healers and health staff [11,56].

Approximately 20% of patients exhibited moderate to severe envenomation, consistent with WHO estimates [10], but lower than the 60% reported from Cameroon [8]. Differences in severity reflect snake species diversity and varying classification criteria, limiting the comparability between studies. Recently, a universal set of core outcome measures and syndrome-specific categories for clinical studies was proposed through a global consensus among stakeholders and expert groups, including statisticians, policy makers, and snakebite patients [57]. A similar consensus-based approach could be extended to community-based surveys, where standardised criteria for classifying severity and syndromes should be established. Existing classification frameworks from previous studies [8,14,30,48,57], as well as the diversity of snake species and their associated syndromes, could guide this work. Such standardisation would improve the comparability of studies and contribute to more accurate assessments of the burden of snakebite.

All snakebite fatalities in this study were female, similar to findings in Nepal [30] and India [58], where deaths were more frequent in females, and Ghana, where an elevated risk of disability was attributed to socio-economic factors [53]. Further, biological factors, such a higher venom-to-body mass ratio, may play a role in this finding [59,60]. However, in Cameroon, mortality was similar between the genders [48] and in Sri Lanka men showed signs of envenomation more frequently [61]. Further research is required to gain a deeper understanding of gender-specific risk factors.

Polyvalent antivenom (Inoserp PAN-AFRICA, Inosan Biopharma, Mexico/Spain) was administered to a high proportion of cases, including those with mild signs of envenomation, where antivenom is generally not required. This raises concerns about inappropriate use, increasing the risk of allergic reactions and imposing unnecessary costs on patients [13,14]. While only one severe allergic reaction was recorded, this may be an underestimation due to incomplete documentation. Additionally. the majority of cases received less than the recommended starting dose of two vials [38], further highlighting inappropriate use of a scarce resource. While Inoserp PAN-AFRICA is reported to be effective against the five medically relevant snake species in Gabon, with at least one study from Cameroon showing its usefulness [62], a risk-benefit assessment of this antivenom by the WHO is ongoing [63].

The use of ‘venom extractors’, common administration of corticosteroids, non-steroidal anti-inflammatory drugs, and antibiotics, with low rates of tetanus vaccinations further demonstrate a lack of snakebite treatment knowledge among health care workers [13,14]. At the time of the study, no national treatment guidelines were available. The development of such guidelines including strict indications for antivenom administration, along with the training of health care workers, are urgently needed to improve the management of snakebites [7,64] and the public’s trust in the health care provided at these facilities.

The estimated median out-of-pocket cost of treatment was 15 times higher in health facilities compared to traditional healers. This disparity is likely due to the high cost of antivenom, which at the time of the survey was USD 132.0 per vial for those with national health insurance and USD 169.4 for those without. Further, transportation costs and poor infrastructure may be barriers to seeking care in formal health facilities, particularly for low-income rural households. Increasing antivenom subsidies, promoting national insurance enrolment, and improving infrastructure could lead more people to seek treatment at health facilities. Initiatives such as motorbike ambulances in Kenya and Nepal have had a positive impact on care-seeking and snakebite mortality [65,66] and could serve as models for Gabon.

Besides direct treatment costs, disabilities and productivity losses or ongoing health care expenses [17,67,68] and the considerable impact on domestic animal populations add to the significant livelihood losses for affected households. In Rwanda, the combined direct treatment costs and indirect productivity losses reach USD 117 per snakebite [68]. In Nepal, snakebite-related mortality among domestic animals resulted in median household losses of USD 90.4, exceeding the losses from health care costs and productivity for snakebites in humans (USD 67.7) [17]. To reduce the burden of snakebites on both animals and humans, preventive measures, such as keeping animals in tightly netted sheds, controlling rodent populations, removing garbage and food to prevent snakes from entering houses and yards should be implemented [16,48].

However, it is also important to recognise the ecological and potential economic role of snakes as predators of rodent populations, which are a major cause of crop damage among smallholder farmers in Africa [69]. A modelling study in southern Australia estimated the financial benefits of the eastern brown snake predation on rodents to be over USD 500 million annually, with additional environmental benefits such as reduced pesticide use and a reduction in rodent-borne zoonotic diseases [70]. The significant financial implications of snakebite envenoming on the one side and ecological and economic importance of snakes on the other warrant further in-depth analysis that goes beyond the scope of this study.

While our study is the first stringently designed epidemiological survey on snakebite burden in Gabon and provides robust data for the department of Ogooué et des Lacs, it is not without limitations.

First, households in the rural and remote sectors were sampled differently from the population in the urban sector. Specifically, we conducted a complete survey of all households in the rural and remote sectors, ensuring comprehensive coverage despite the absence of an official sampling frame, but performed random sampling in the urban sector. To account for the incomplete sampling in the urban sector, we calculated the standardised incidence rate, which considers the differences in the proportion of the population sampled in each sector, which we believe provides a reliable estimate of snakebite incidence in the department.

Second, our results may not be generalisable to other areas of Gabon where the differences in population density, economic activity, landscape, snake fauna, and climate may result in differences in snakebite burden. However, this study provides the first estimate of the snakebite burden in Gabon, which was higher than expected and indicates the need for further research in other areas of Gabon and other countries in Central Africa.

Third, supplementary respondents may have been more likely to approach the study team to report severe cases than mild cases, leading to a possible overreporting of severe envenomations. However, the estimated proportion of cases classified as severe aligns with WHO estimates and does not exceed what has been reported elsewhere [8,10,71].

Finally, the ability to assign a snake species based on descriptions was limited, as described previously [72]. Developing visual aids with photographs of local species could help snakebite victims in identifying snake species, facilitating the identification of commonly involved snake species and informing educational materials for health care workers and community members. Recently, automated identification technology supported by artificial intelligence has been successfully demonstrated for snakes [73] and could be implemented in mobile apps to improve species identification by community members and health care professionals.

Despite these limitations, many of which are inherent to all cross-sectional studies on snakebites, we provide a comprehensive assessment of the burden of snakebite in Ogooué et des Lacs, highlighting the importance of snakebite envenoming in Gabon and the need for further epidemiological studies across Africa to close the data gap and improve our understanding of the burden of this deadly neglected tropical disease on the African continent [74,75].

## CONCLUSIONS

This comprehensive epidemiologic study highlights the considerable impact of snakebites in Gabon and has contributed to snakebite envenomation being included in Gabon’s National Health Development Plan for 2024 to 2028 [43]. To effectively reduce the burden of snakebite envenoming in Gabon, the planning and implementation of specific interventions and strategies are essential. Priority interventions should include community engagement programmes to promote prevention strategies and encourage effective first-aid methods and appropriate treatment-seeking behaviour; training of health care workers in the management of snakebites and the development treatment guidelines; the provision and subsidy of effective antivenom; and the development of collaboration strategies with traditional healers.

## Additional material


Online Supplementary Document
