# Supplementary material for: The epidemiology of snakebites, treatment-seeking behaviour, and snakebite management in the department of Ogooué et des Lacs, Gabon, Central Africa: a cross-sectional community and health facility-based survey
Source: J Glob Health. 2025 Apr 25;15:04062. doi: 10.7189/jogh.15.04062 (PMC12023806; doi:10.7189/jogh.15.04062)
Supplement: Online Supplementary Document [file jogh-15-04062-s001.pdf]

Supplement to: Artus R, Rae J, Hunstig F, Mombo-Ngoma G, Houmenou Zinsou A, Okwu DG, Ndzebe Ndoumba W, Zoleko Manego R, Ramharter M, Lell B, Kremsner PG, Aron MB, Blessmann J, Kreuels B. The epidemiology of snakebites, treatment-seeking behaviour, and snakebite management in the department of Ogooué et des Lacs, Gabon, Central Africa: a cross-sectional community and health facility-based survey. J Glob Health. 2025;15:04062.

Contents

Page 1-2: STROBE checklist.

Page 3-7: Community questionnaire.

Page 8: Table S1. Classification of snakebite severity and syndromes.

Page 19: Figure S1. Flowchart illustrating the inclusion of households in the cross-sectional community survey.

Page 10: Table S2. Characteristics of snakes seen following snakebites to humans and animals.

Page 11: Table S3. Classification of symptoms for snakebite cases where a possible snake species could be assigned.

Page 12: Table S4. The first health provider visited following a snakebite by sector and envenomation severity

The Strengthening the Reporting of Observational Studies in Epidemiology (STROBE) statement: guidelines for reporting observational studies

	Item No	Recommendation	Page No
**Title and abstract**	1	(*a*) Indicate the study's design with a commonly used term in the title or the abstract	1-3
		(*b*) Provide in the abstract an informative and balanced summary of what was done and what was found	3
**Introduction**			
Background/rationale	2	Explain the scientific background and rationale for the investigation being reported	4-5
Objectives	3	State specific objectives, including any prespecified hypotheses	5
**Methods**			
Study design	4	Present key elements of study design early in the paper	5
Setting	5	Describe the setting, locations, and relevant dates, including periods of recruitment, exposure, follow-up, and data collection	5-7
Participants	6	(*a*) Give the eligibility criteria, and the sources and methods of selection of participants	6-7
Variables	7	Clearly define all outcomes, exposures, predictors, potential confounders, and effect modifiers. Give diagnostic criteria, if applicable	7-9
Data sources/ measurement	8	For each variable of interest, give sources of data and details of methods of assessment (measurement). Describe comparability of assessment methods if there is more than one group	8-9
Bias	9	Describe any efforts to address potential sources of bias	6-8
Study size	10	Explain how the study size was arrived at	6-7
Quantitative variables	11	Explain how quantitative variables were handled in the analyses. If applicable, describe which groupings were chosen and why	8-9
Statistical methods	12	(*a*) Describe all statistical methods, including those used to control for confounding	8-9
		(*b*) Describe any methods used to examine subgroups and interactions	N/A
		(*c*) Explain how missing data were addressed	9
		(*d*) If applicable, describe analytical methods taking account of sampling strategy	6-7
		(*e*) Describe any sensitivity analyses	N/A
**Results**			
Participants	13	(a) Report numbers of individuals at each stage of study—eg numbers potentially eligible, examined for eligibility, confirmed eligible, included in the study, completing follow-up, and analysed	10, 13
		(b) Give reasons for non-participation at each stage	10
		(c) Consider use of a flow diagram	Figure S1

Descriptive data	14	(a) Give characteristics of study participants (eg demographic, clinical, social) and information on exposures and potential confounders	10-13
		(b) Indicate number of participants with missing data for each variable of interest	10-14
Outcome data	15	Report numbers of outcome events or summary measures	10,13
Main results	16	(*a*) Give unadjusted estimates and, if applicable, confounder-adjusted estimates and their precision (eg, 95% confidence interval). Make clear which confounders were adjusted for and why they were included	Table 1 & 4
		(*b*) Report category boundaries when continuous variables were categorized	N/A
		(*c*) If relevant, consider translating estimates of relative risk into absolute risk for a meaningful time period	N/A
Other analyses	17	Report other analyses done—eg analyses of subgroups and interactions, and sensitivity analyses	N/A
**Discussion**			
Key results	18	Summarise key results with reference to study objectives	14-15
Limitations	19	Discuss limitations of the study, taking into account sources of potential bias or imprecision. Discuss both direction and magnitude of any potential bias	19-20
Interpretation	20	Give a cautious overall interpretation of results considering objectives, limitations, multiplicity of analyses, results from similar studies, and other relevant evidence	14-19
Generalisability	21	Discuss the generalisability (external validity) of the study results	14-15
**Other information**			
Funding	22	Give the source of funding and the role of the funders for the present study and, if applicable, for the original study on which the present article is based	21

von Elm E, Altman DG, Egger M, Pocock SJ, Gøtzsche PC, Vandenbroucke JP. The Strengthening the Reporting of Observational Studies in Epidemiology (STROBE) statement: guidelines for reporting observational studies. PLoS Med. 2007;4:e296.

Community Questionnaire

Household information

1. Household ID _____________
2. How many people have lived in the household for ≥ 6months in the previous year? _____________
3. Have any of these people ever been bitten by a snake?
   - o Yes
   - o No
4. Is there a child between the ages of five and ten living in this household?
   - o Yes
   - o No

   4.1 If yes, how old is one of these children now? _____________
5. Have you owned animals in the previous 12 months in your household?
   - o Yes
   - o No

   5.1 If yes, which type of animal?
   - o Cow
   - o Dog, specify the number of dogs _____________
   - o Sheep
   - o Goat
   - o Cat, specify the number of cats _____________
   - o Other, specify ____________

   5.2 Has an animal been bitten by a snake in the previous 12 months?
   - o Yes
   - o No

   5.2.1. If yes, which type of animal was bitten?
   - o Chicken
   - o Dog
   - o Sheep
   - o Goat
   - o Cat
   - o Other, specify____________

   5.2.2. How many animals were bitten (in total)? ____________

   5.2.3. Did someone see the snake?
   - o Yes
   - o No

   5.2.4. What was the colour of the snake responsible for the bite?
   - o Green
   - o Black
   - o Brown
   - o Do not know
   - o Other, specify____________

   5.2.5. Is the name of the snake known?
   - o Yes, specify name ____________
   - o No

   5.2.6. What was the outcome after the snakebite?
   - o Full recovery
   - o Sequelae or disability, specify____________
   - o Death
6. Did you (the person who signed the consent) have a tick bite in the previous 12 months?
   - o Yes
   - o No

   6.1 If yes, how many____________
7. Interviewer ____________
8. Date of the interview (dd/mm/yyyy)____________
9. Informed consent given

- o Yes
- o No

10. Remarks___________________________

Household member (HM)/ Individual information

1. Age____________
2. Sex
   - o Male
   - o Female
3. Has the person ever been bitten by a snake?
   - o Yes
   - o No*

**If no, repeat questions 1 to 3 (HM) with the next HM. If yes, the following questions will be posed:*

Information on the snakebite

1. Year of snakebite (yyyy) ____________
2. Month of snakebite (mm)____________
3. In which season did the bite occur?
   - o Rainy
   - o Dry
4. When did the snakebite happen?
   - o Last year
   - o >1 & ≤5 years ago
   - o > 5 years ago

Clarifying question (CQ): How many dry seasons have passed since the incident. How old was a child in the household when the snakebite happened and how old is the child now? Age at the event and age now - is it congruent with time? Did anyone witness the event? If available, can they confirm the event and time?)

5. Age at snakebite____________
6. Did the snakebite happen in the department of Ogooué et des Lacs?
   - o Yes
   - o No, where? ____________
7. Where did the bite occur?
   - o In the house
   - o In the garden or yard of your house
   - o In the field
   - o In the forest
   - o On a footpath
   - o On the shore
   - o Other, specify ____________
8. During what kind of activity did the bite occur? ____________
9. The time period of bite
   - o Morning (6:00 - 11:59)
   - o Afternoon (12:00 - 16:59)
   - o Evening (17:00 to 20:59)
   - o Night (21:00 to 5:59)
   - o Not indicated
10. Who saw the snake?
    - o Interview partner
    - o Somebody
    - o Nobody

CQ: Were there circumstances that made it difficult to see to clearly identify the snake, darkness, underwater, ran away?

CQ: Were there bite marks/symptoms typical for a snake?

10.1 If the snake was seen, what was the colour of the snake?
- o Green
- o Black
- o Brown
- o Do not know
- o Other, specify:____________

10.2 What size was the snake? (*Show the approximate size)*
- o >30cm
- o 30cm-1m
- o >1m

10.3 Is the name of the snake known?
- o Yes, specify the name ____________
- o No

11.Where did the snake bite?
- o Lower limb
- o Upper limb
- o Trunk
- o Head/Neck

12.Symptoms of envenomation (multiple possible):

12.1 Swelling at the bite site

12.1.1. Severity of swelling
- o Mild
- o Moderate
- o Severe

12.1.2. Duration of swelling
- o < 1 hour
- o < 1 day
- o < 1 week
- o < 1 month
- o < 6 month
- o < 1 year
- o > 1 year

12.2 Bleeding, specify____________

12.2.1. Severity of bleeding
- o Mild
- o Moderate
- o Severe

12.2.2. Duration of bleeding
- o < 1-hour
- o < 1-day
- o < 1-week
- o < 1-month
- o < 6 months
- o < 1-year
- o > 1-year

12.3 Ptosis or other neurological signs, specify ____________

12.3.1. The severity of ptosis or other neurological signs
- o Mild
- o Moderate
- o Severe

12.3.2. Duration of ptosis or other neurological signs
- o < 1 hour
- o < 1 day
- o < 1 week
- o < 1 month
- o < 6 month

- < 1 year
- > 1 year

12.4 Pain

12.4.1. Severity of pain
- Mild
- Moderate
- Severe

12.4.2. Duration of pain
- < 1 hour
- < 1 day
- < 1 week
- < 1 month
- < 6 month
- < 1 year
- > 1 year

12.5 Other (nausea, vomiting, abdominal pain)__________________

13. Where did you go for treatment after the snakebite – Place of treatment (*Order of treatment-seeking must be defined, only one option per column is possible*)

	**Row of treatment-seeking**				
**Treatment place**	1	2	3	5	6
Hospital					
Pharmacy/ primary health facility					
Traditional healer					
Self-treatment					
Nowhere					
Other					

13.1 If traditional healer, specify ____________

13.2 If a hospital, provide the name of hospital ____________

14. What kind of treatment did you get?
- Antivenom
- Analgesia
- Antibiotics
- Blood transfusion
- Herbs
- No treatment
- Wound treatment
- Do not remember
- Other, specify ____________

15. Did you have to pay for the treatment?
- Yes, specify the approximate amount ____________
- No

16. What was the outcome after the snakebite?
- Full recovery
- Sequelae or disability (≥ 6 months), specify_________________________

16.1 If yes, did the sequelae keep you away from work?
- Yes, specify duration ______________
- No

16.2 Did the sequelae from the bite keep you from doing your household chores?
- Yes, specify duration__________________
- No

16.3 Did the symptoms after the bite disturb your sleep?
- Yes, specify how
- No

17. Were you scared after the bite?

- o Yes
- o No

17.1 If yes, were you disturbed in your daily life by the fear?

- o Yes
- o No

18.Do you have scars from the snakebite?

- o Yes
- o No

18.1 If yes, were you ashamed due to them?

- o Yes
- o No

18.2 Did they keep you from joining community activities or meeting family or friends?

- o Yes
- o No

19.Is the snakebite plausible?

- o Yes
- o No

CQ: Who came to the conclusion of snakebite? Did you discuss this with others (family, teachers, colleagues in the field, etc.)
CQ: Are there any factors about the individual (dementia, alcohol or drug abuse, mental impairment, impaired vision) that make the interviewer doubt the precise and correct answer? If yes, is there a reliable person who can confirm what happened?

20. Occupation of the person?

- o Housewife
- o Fisher
- o Agricultural worker
- o Business/ Trade
- o Student
- o Driver
- o Children
- o Unemployed
- o Forester
- o Hunter
- o Other. Specify____________

21.Has the person ever been bitten by a dog?

- o Yes
- o No

21.1 Year of the dog bite (yyyy)____________

21.2 When did the dog bite happen?

- o Last year
- o >1- ≤5 years ago
- o > 5 years ago

21.3 Is the bitten person still alive?

- o Yes
- o No

21.3.1. If no, how much time passed between the bite and the death

- o < 1 month
- o < 3 months
- o < 12 months/ 1 year
- o > 12 months/ 1 year
- o Don’t know

22. Remarks____________________________________

Table S1. Classification of snakebite severity and syndromes

Severity grade	Syndrome	Signs and symptoms*
**Dry†/Mild**	N/A	No signs or symptoms, or none of the symptoms listed below
**Moderate**	N/A	Vomiting, loss of consciousness, no severe signs
**Severe‡**	Cytotoxic	Pain and severe swelling§ around the bite site with blisters or necrosis
	Neurotoxic	Paralysis of the affected limb, difficulty opening eyes, difficulty walking, difficulty swallowing or breathing
	Haematotoxic	Persistent bleeding from the bite wound, spontaneous bleeding, reported blood in stool or vomitus

*Signs and symptoms within each classification could occur alone or in combination.

†Snakebite without signs of envenomation.

‡A combination of severe syndromes was possible. Individuals who died as a result of the snakebite were classified as severe, including cases where symptoms were not reported.

§Pain and swelling were self-reported as severe, moderate or mild. The progression of swelling was not collected.

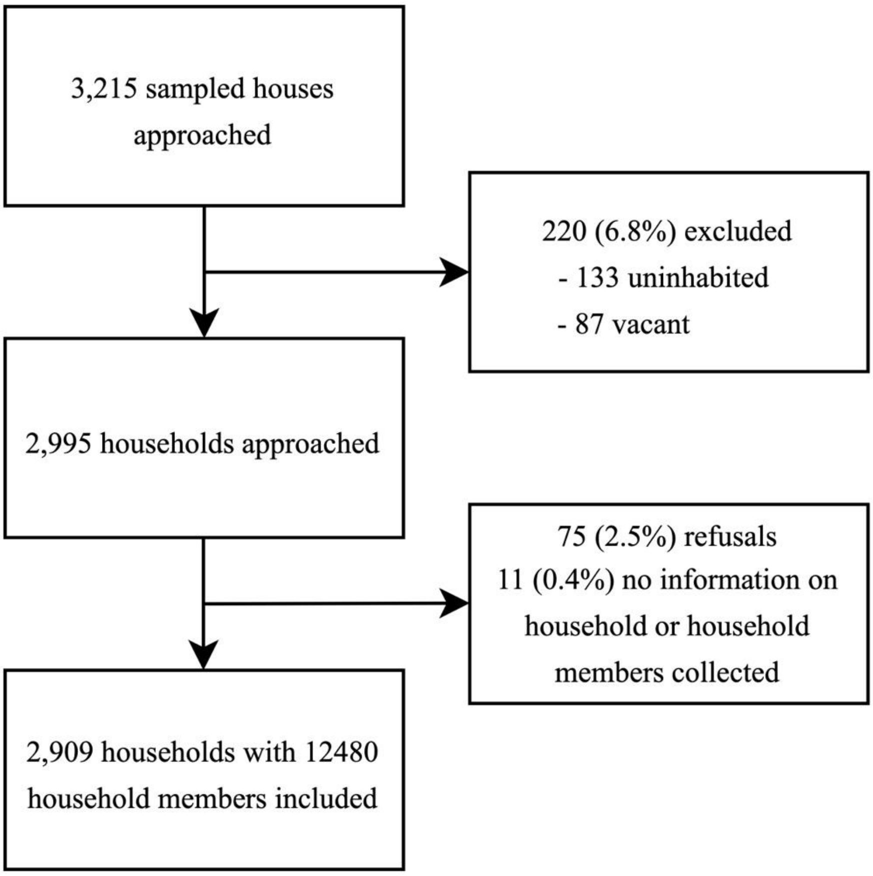

Figure S1. Flowchart illustrating the inclusion of households in the cross-sectional community survey.

Table S2. Characteristics of snakes seen following snakebites to humans and animals

**Characteristic**	**Snakebite to humans**	**Snakebites to animals**
	**% (n/N)**	
Snake seen after bite	841 (148/176)	100 (450/450)
**Reported snake colour**	91.9 (136/148)	98.4 (443/450)
**Unicoloured**		
1. Black	27.9 (38)	56.4 (250)
2. Brown	9.6 (13)	5.2 (23)
3. Green	8.8 (12)	0.5 (2)
4. Grey	2.9 (4)	0.7 (3)
5. Yellow	1.5 (2)	0.2 (1)
**Multicolour**		
6. Brown & beige/camouflage/yellow	11.8 (16)	5.0 (22)
7. Black & yellow (spots, bands or venter)	11.0 (15)	24.2 (107)
8. Grey & beige/camouflage/yellow	5.1 (7)	4.5 (20)
9. Green & yellow	2.2 (3)	0.0 (0)
10. Other combinations	19.1 (26)	3.4 (15)
**Reported snake name***	70.3 (104/148)	93.8 (422/443)
a. Serpent noir (black snake)/Moudouma†/Evini-nyong‡/Iwombia§	34.6 (36)	73.2 (309)
b. Python	14.4 (15)	17.8 (75)
c. Vipère (viper)	10.6 (11)	0.7 (3)
d. Duere/-a‖	6.7 (7)	0.0 (0)
e. Mamba vert/jaune (green/yellow mamba)	4.8 (5)	0.0 (0)
f. Mamba noir (black mamba)	4.8 (5)	2.4 (10)
g. Serpent de l'eau (water snake)	1.9 (2)	0.0 (0)
h. Cobra	1.0 (1)	0.9 (4)
i. Other names	21.2 (22)	5.0 (21)
**Possible snake species¶**.	13.1 (23/176)	30.0 (135/450)
Forest cobra (*N. melanoleuca*) (6 & a/f/h)	39.1 (9)	69.6 (94)
Seba Python (*P. sebae*) (6/8 & b)	34.8 (8)	29.6 (40)
Gaboon viper (*B. gabonica*) (6 & c)	26.1 (6)	0.7 (1)

*Respondents reported names in free-text sections (in French or local languages), resulting in snakes not limited to those typically found in the study area.

†‡§Names in Punu†, Fang‡, and Myènè§ languages translate to "black snake" and refer to *N. melanoleuca* (Pauwels OSG. Slippery customers. Gabon Magazine. 2009:34-7, Pauwels OSG, Vande weghe JP. Animaux dangereux et reputes dangereux du Gabon. Libreville: Shell Gabon; 2013, Pauwels OSG).

‖Name in Punu and Sira languages for "green snake".

¶Based on the reported colours and names consistent with common species in the study area
(Pauwels OSG, Vande weghe JP. Animaux dangereux et reputes dangereux du Gabon. Libreville: Shell Gabon; 2013, Les reptiles. In: Vande weghe JP, Stévart T, editors. Le Delta de l'Ogooué. Libreville, Gabon: Agence Nationale des Parcs Nationaux; 2017. p. 256–65).

Table S3. Classification of symptoms for snakebite cases where a possible snake species could be assigned

Assigned snakes	Forest cobra (*N. melanoleuca*)	Seba python (*P. sebae*)	Gaboon viper (*B. gabonica*)
	**N; % (n)**		
Number of cases*	9	8	6
Dry/mild	66.7 (6)	100 (8)	83.3 (5)
Moderate	11.1 (1)	0.0 (0)	0.0 (0)
Severe	22.2 (2)	0.0 (0)	16.7 (1)
**Syndromes of severe envenomation**			
Cytotoxic	0.0 (0)	0.0 (0)	16.7 (1)
Neurotoxic	22.2 (2)	0.0 (0)	0.0 (0)
Neurotoxic and cytotoxic	0.0 (0)	0.0 (0)	0.0 (0)
Unclear	0.0 (0)	0.0 (0)	0.0 (0)

Classification of envenomation was done retrospectively using the criteria detailed in Table S1.
*The number of cases where possible snake species is assigned, as detailed in Table S2.

Table S4. The first health care provider visited following a snakebite by sector and envenomation severity

	**Sector, n/N (%)**			**Classification of snakebite severity, n/N (%)***			
	**Urban**	**Rural**	**Remote**	**Mild**	**Moderate**	**Severe**	**Total**
Health facility	21/26 (80.8)	47/89 (52.8)	28/60 (46.7)	71/142 (50.0)	13/18 (72.2)	12/14 (85.7)	96/175 (54.9)
Traditional healer	2/26 (7.7)	23/89 (25.8)	14/60 (23.3)	33/142 (23.2)	4/18 (22.2)	1/14 (7.1)	39/175 (22.3)
No consultation	3/26 (11.5)	19/89 (21.3)	18/60 (30.0)	38/142 (26.8)	1/18 (5.6)	1/14 (7.1)	40/175 (22.9)

*Information on severity and treatment-seeking was missing for one snakebite case. Severity was determined retrospectively based on the criteria detailed in Table S1.
